# Analytical and Clinical Validation of a New Immunoenzymatic Method for the Measurement of Canine Parathyroid Hormone

**DOI:** 10.3390/ani10122411

**Published:** 2020-12-17

**Authors:** Jari Zambarbieri, Filippo Tagliasacchi, Pierangelo Moretti, Alessia Giordano, Paola Scarpa

**Affiliations:** Department of Veterinary Medicine, University of Milan, Via dell’Università 6, 26900 Lodi, Italy; filippotagliasacchi@gmail.com (F.T.); pierangelo.moretti@unimi.it (P.M.); alessia.giordano@unimi.it (A.G.); paola.scarpa@unimi.it (P.S.)

**Keywords:** parathyroid hormone, dog, method validation, biological marker, chronic kidney disease, renal hyperparathyroidism

## Abstract

**Simple Summary:**

In dogs affected with chronic kidney disease (CKD), mineral disorders, including renal hyperparathyroidism (RHPT), are frequent. Secondary RHPT is the increase in serum parathyroid hormone (PTH), that can have a significant impact in the disease progression. Despite its clinical utility, the measurement of serum PTH is not routinely executed due to limited availability of validated methods. The aims of this study were: the analytical validation of a new method for PTH measurement in dogs and analysis of the preliminary association of the obtained results with the renal status. Twenty-seven samples obtained from dogs that were healthy or affected with CKD were analysed. PTH was measured using a commercially available human assay. The precision and accuracy of this method were assessed and the PTH stability at different temperatures was evaluated. Clinical validation was performed by comparing PTH values with clinicopathological parameters often altered during CKD, such as creatinine and phosphorus, and with the disease severity. The method showed an optimal precision and accuracy; the stability was compatible with the standard sample processing times. PTH was positively associated with creatinine and phosphorus. The investigated method was successfully validated in dogs, allowing its use for clinical purposes.

**Abstract:**

Renal hyperparathyroidism (RHPT) is one of the main complications in dogs affected with Chronic Kidney Disease (CKD). The measurement of serum parathyroid hormone (PTH) could be of clinical utility for the disease’s treatment and follow-up; however, PTH is not routinely determined due to limited available methods, often not fully validated in dogs. The aims of this study were the analytical validation of an immunoenzymatic method for the measurement of PTH in canine serum and the analysis of preliminary association of the obtained results with renal function. Twenty-six samples obtained from dogs healthy or affected with CKD were analysed. PTH was measured using a two-site immunoenzymometric human assay (ST AIA-PACK^®^ Intact PTH, Tosoh Bioscience). The analytical validation protocol evaluated the assay precision and accuracy. Also, the PTH’s storage stability at 20 °C, 4 °C and −20 °C was assessed. Clinical validation was performed by comparing PTH values with creatinine, phosphorus and International Renal Interest Society (IRIS) stage. The method showed optimal precision and accuracy, whereas stability was adequate up to 4 h at 20 °C, 24 h at 4 °C and 6 months at −20 °C. PTH was positively associated with creatinine, phosphorus and IRIS stage. The investigated method was thus successfully validated in dogs, allowing its use for clinical purpose.

## 1. Introduction

Parathyroid hormone (PTH) is a single chain 84-amino acid polypeptide produced from parathyroid glands and primarily involved in the regulation of circulating ionized calcium (iCa) concentrations. PTH is highly conserved among mammalian species, with 88% of homology between human and canine PTH [1].

In patients affected with chronic kidney disease (CKD), serum PTH increases due to a complex pathophysiological mechanism involving phosphorus (P), Fibroblast Growth Factor−23 (FGF-23), vitamin D metabolites and iCa [2]. Renal secondary hyperparathyroidism (RHPT) is a major complication of CKD in dogs, which has an increasing prevalence according to the International Renal Interest Society (IRIS) CKD staging system: the prevalence increases from 36% in stage 2 to 100% in stage 4 in dogs and in some cases it is prior to the presence of hyperphosphatemia [3]. Other studies showed the decrease of several vitamin D metabolites in dogs affected with CKD, and a negative correlation between vitamin D metabolites and PTH, FGF-23, and P concentrations, supporting the role of these hormones in the development of RHPT [4,5].

In this scenario the routine measurement of PTH would allow early detection of RHPT; establishment of adequate treatments, such as calcitriol administration; verification of treatments effectiveness; and monitoring of the disease progression. However, even if recommended, PTH measurement is not routinely performed due to the limited availability of fully validated diagnostic tests in dogs and the high execution costs. In fact, despite several different assays being reported in published studies, data about their validation are not always exhaustive, as reported below in details [3,4,6,7,8].

Frequently assays successfully employed in veterinary medicine are humans or derived from human ones; however, before their use for clinical purpose a validation protocol in the species of interest is always necessary. Different generation of assays for the measurement of human PTH have been reported. Briefly, first-generation PTH assays used a single polyclonal antibody directed against the PTH C-terminal part or midterminal part; these assays are currently abandoned because they used to measure many biologically inactive C-terminal fragments affecting the test specificity. Second-generation assays, which include all the methods used in veterinary medicine, are known as intact PTH assays and, thanks to an extra antibody, they should measure only full-length PTH. However, these assays were reported to measure also C-terminal fragments. A third-generation assays, known as whole or bio-intact PTH assay, able to detect only full-length PTH has been recently developed [9].

Regarding methods used to measure canine PTH, some fully validated immunoradiometric assays (IRMA), such as Allegro Intact PTH (Nichols Institute Diagnostics, San Juan Capistrano, CA, USA), used in some studies, are no longer available [10,11,12,13,14,15]. Moreover, a dual whole and intact human IRMA (Duo PTH, Scantibodies, Santee, CA, USA) has been sufficiently validated, but radioimmunoassays are not available in all veterinary laboratories [6,16]. A whole PTH assay offered by Michigan State University Diagnostic Center for Population and Animal Health is only partially validated [4,7,17,18]. A canine enzyme-linked immunosorbent assay or ELISA (Canine intact PTH, Immutopics, San Clemente, CA, USA) is widely used even in the absence of a complete validation for the canine species [8]; a chemiluminescent intact PTH assay for human use (Immulite intact PTH, Siemens Healthcare Medical Solutions Diagnostics, Erlangen, Germany) is partially validated and not recommended for use in dogs, even if able to detect high PTH values in CKD dogs [3,16]. A point of care similar assay (Immulite Turbo Intact PTH, Diagnostic Products Corporation, Los Angeles, CA, USA) is fully validated but no longer suitable for use in dogs [19,20]. Other ELISA kits for detection of intact PTH such as Canine Intact PTH EIA MicroVue Bone (Quidel Corporation, San Diego, CA, USA), Canine PTH ELISA kit (Elisagenie, London, UK) or Dog PTH ELISA kit competitive EIA (LifeSpan BioSciences, Seattle, WA, USA) are available for research use only with different information about precision, accuracy, sensitivity, stability and limit of detection, which is about 3 pg/mL. According to this complex scenario, PTH values obtained in dogs could be unreliable or change significantly based on the method chosen.

The aims of this study were: the analytical validation of a new immunoenzymatic method validated in humans for the measurement of PTH in canine serum and the analysis of the preliminary association of the obtained results in light of the canine patients’ renal function.

## 2. Materials and Methods 

### 2.1. Animals

This study was carried out including 26 samples obtained from 20 dogs healthy or affected with CKD, referred to the University of Milan Veterinary Teaching Hospital during the routine clinical activity between March and September 2019. Inclusion criteria for the enrollment of dogs in the healthy group were: absence of clinical signs and recent history of disease, and absence of laboratory changes. CKD dogs were included according to IRIS guidelines: serum creatinine persistently above 1.4 mg/dL and/or presence of renal proteinuria (urinary protein:creatinine ratio higher than 0.5) and/or presence of ultrasonographic abnormalities compatible with CKD. All nephropathic dogs included in the study were staged according to the International Renal Interest Society (IRIS) guidelines (www.iris-kidney.com). Three dogs were sampled more than one time basing on the IRIS staging progression or worsening of general condition.

All dogs undergoing physical examination according to standard veterinary procedures and blood withdrawal for diagnostics purposes were sampled after obtaining the owner’s consent. According to the Ethics Committee of the University of Milan (EC decision 29 October 2012, renewed with the protocol no. 02-2016), biological samples collected in these setting could be used also for research purposes and a formal approval was not required. Two to five mL of blood were collected from the cephalic vein and placed into methacrylate tubes without anticoagulants, pre-filled with a gel separator and clot activator (FL Medical, Torreglia, Padua, Italy), followed by centrifugation (10 min, 2500× *g*) within 30 min from collection and execution of routine analyses within 2 h. The leftover serum was employed for the measurement of PTH.

### 2.2. PTH Measurement

The measurement of PTH was performed after routine analyses using an automated analyzer (AIA 360^®^) and a two-site immunoenzymometric assay (ST AIA-PACK^®^ Intact PTH, Tosoh Bioscience, Tessenderlo, Belgium) performed entirely in the test cups. Briefly, intact PTH present in the sample is bound with polyclonal antibody immobilized on magnetic solid phase and enzyme-labeled polyclonal antibody. The magnetic beads are washed to remove unbound enzyme-labeled polyclonal antibody and are then incubated with the fluorogenic substrate 4-methylumbelliferyl phosphate (4MUP). The amount of enzyme-labeled polyclonal antibody that binds to the beads is directly proportional to the intact PTH concentration in the sample. A standard curve is constructed, and unknown sample concentrations are calculated using this curve. The standard curve was obtained using six calibrators provided by the manufacturer with increasing concentrations of PTH: 0 pg/mL, 16.58 pg/mL, 47.90 pg/mL, 195.78 pg/mL, 757.5 pg/mL, and 2194.6 pg/mL. The calibration curve is validated for 90 d. Additionally, before each work session, three multi analyte control sera with established concentrations (10.70 pg/mL, 33,60 pg/mL, 213.50 pg/mL) were used and the result was accepted if within 2 standard deviations.

### 2.3. Creatinine and Phosphorus Measurement

Biochemical analysis was conducted using a fully automated spectrophotometric analyzer (BT 3500, Biotecnica Instruments, Rome, Italy) and a colorimetric kinetic modified Jaffé method for serum creatinine (sCr) and the phosphomolybdate UV method for phosphorus (P) with reagents provided by Futurlab Srl (Limena, Italy).

### 2.4. Analytical Validation

The following validation steps were performed using calibrators, control materials and reagents provided by the manufacturer, which are all human based. All the pooled sera were obtained by merging specimen from dogs included in the study and grouped based on their PTH concentration.

#### 2.4.1. Precision and Accuracy

The intra-assay imprecision was determined by measuring PTH in canine pooled sera with low (3.52 pg/mL), medium (29.13 pg/mL) and high (95.15 pg/mL) PTH levels; 5 replicates of each measurement within a single run of analysis were done on each pool. The inter-assay variability was assessed by analyzing the same samples, in duplicate, on 5 consecutive working days. Mean value, Standard Deviation (SD) and Coefficient of Variation (CV = SD/mean × 100) were calculated. 

The accuracy was determined using the evaluation of linearity under dilution (LUD) and a spik-recovery test (SRT): LUD was performed by measuring PTH in triplicate on a pool of canine sera with high PTH concentration (89.2 pg/mL) after serial dilutions with deionized water to obtain solutions containing 90%, 80%, 70%, 60%, 50%, 40%, 30%, 20% and 10% of the serum sample, respectively. In absence of an accepted gold standard to measure canine PTH, nor of an available purified canine PTH, SRT was performed by mixing a pool with low PTH level (3.5 pg/mL) with increasing percentages (10% to 100%) of a pool with high PTH level (73.9 pg/mL) followed by a measurement in triplicate. The association between the obtained and the expected values in the LUD and SRT tests was assessed using a least squares regression. 

Lower Limit of Detection (LLOD) of the method was obtained using the following formula: LLOD = blank + 1.645(SD_low concentration sample_), where blank is the calibrator with a concentration of 0 pg/mL and low concentration sample is the mean of the lowest values obtained by LUD [21].

The blank was tested using the calibrator 1 provided by the manufacturer, which is described to contain 0 pg/mL.

#### 2.4.2. Storage Stability

Storage stability was evaluated using fresh canine sera, which have been divided in low and medium pools (7.87 pg/mL and 27.63 pg/mL, respectively) analyzed immediately after sampling in triplicate and then stored at room temperature (about 20 °C), refrigeration temperature (4 °C) and freezing temperature (−20 °C). They were re-analyzed in triplicate after the following times: 4 h, 8 h and 24 h at room temperature; 24 h, 48 h and 72 h at refrigeration temperature; 1 week, 1 month and 6 months at freezing temperature. For the analysis of the obtained results, two different cut-off values of tolerance were established: the intra-assay CV of 7% (based on the CV obtained in the assessment of precision) and a deviation of ±10% from the mean baseline value, as reported by previous validation studies [22].

### 2.5. Clinical Validation

Clinical validation was performed by comparing PTH values with sCr, P and IRIS stage. 

Statistical analysis was performed with the software JMP 14 (SAS Inc., Cary, NC, USA) including a descriptive analysis and the following tests: Shapiro-Wilk test was used to assess the normality of the data; linear regression for not parametric data for the analysis of association between PTH and sCr, and PTH and P. Kruskal-Wallis test was used to compare PTH concentrations by IRIS stages, while Wilcoxon-Mann Whitney test was used as a post-hoc; since the low number of samples from each stage, the samples were pooled in three groups: 0 (healthy dogs), 1–2 and 3–4.

Based on the pathophysiology of RHPT as reported above, a positive association was expected between PTH and both sCr and P.

## 3. Results

### 3.1. Animals

Twenty dogs were included in the study: a total of 5 dogs were healthy, and 15 dogs were affected with different stages of chronic kidney disease: 2 dogs in IRIS stage 1, 8 in IRIS stage 2, 4 in IRIS stage 3 and 1 in IRIS stage 4. 

Healthy dogs were: 2 Mongrels, 1 Springer Spaniel, 1 Great Dane and 1 Cocker Spaniel; there were 2 neutered males, 1 male and 2 spayed females. Affected dogs were: 4 Mongrels, 3 Golden Retriever, 2 Labrador Retriever, 1 Boxer, 1 Fox Terrier, 1 German Shepherd, 1 Maltese and 1 Welsh Corgi Pembroke; there were 13 females (of which 7 spayed) and 2 males (of which 1 neutered). The age ranged from 1 to 16 years with a median of 6 years. Complete data are reported in Table S1.

### 3.2. Precision and Accuracy

The results of the intra- and inter-assay precision assessment evaluated in pooled sera with low, medium and high PTH concentration are reported in Table 1. The CVs were <7% for all three levels of PTH; in both tests, a higher imprecision was found in the low pool.

**Figure 1:**
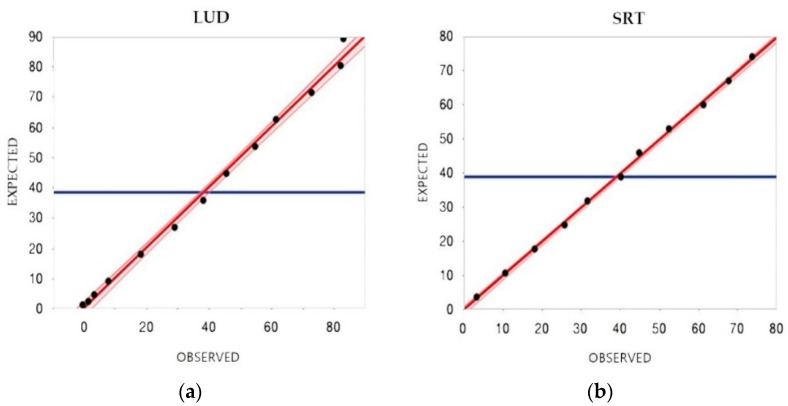
Comparison between observed and expected results: (**a**) Linearity under dilution (LUD) of PTH concentration in a pool of sera serially diluted with distilled water. (**b**) Spike-recovery test (SRT) of PTH concentration in a pool of sera with low PTH concentration spiked with increasing amounts of a serum pool with high PTH concentration. The solid line indicates the linear association between expected and observed results. The blue line indicates the expected mean.

The results obtained after LUD and SRT are reported in Figure 1. A comparison between expected and obtained reasults of the SRT is reported in Table 2. Both tests fitted the linear model (*r*^2^ = 0.99, *p* < 0.001 for the LUD test; *r*^2^ = 1, *p* < 0.001 for the SRT) showing an optimal agreement between observed and expected results. This part of the study allowed the determination of the LLOD of the method, which was about 1.3 pg/mL.

### 3.3. Storage Stability

**Figure 2:**
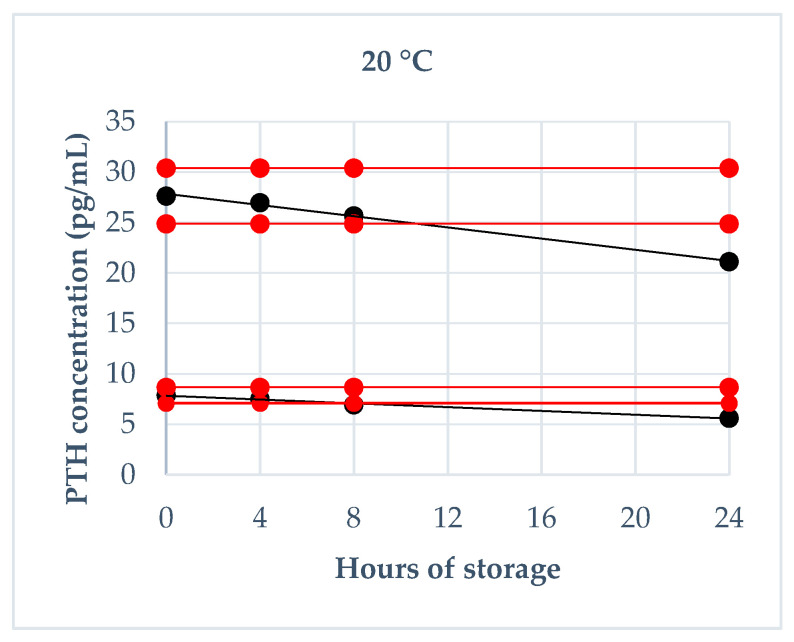
PTH stability at 20 °C during 24 h of storage (4 h, 8 h and 24 h): the black lines indicate the tendency of the obtained values; the red lines indicate ± 10% from the baseline value.

**Figure 3:**
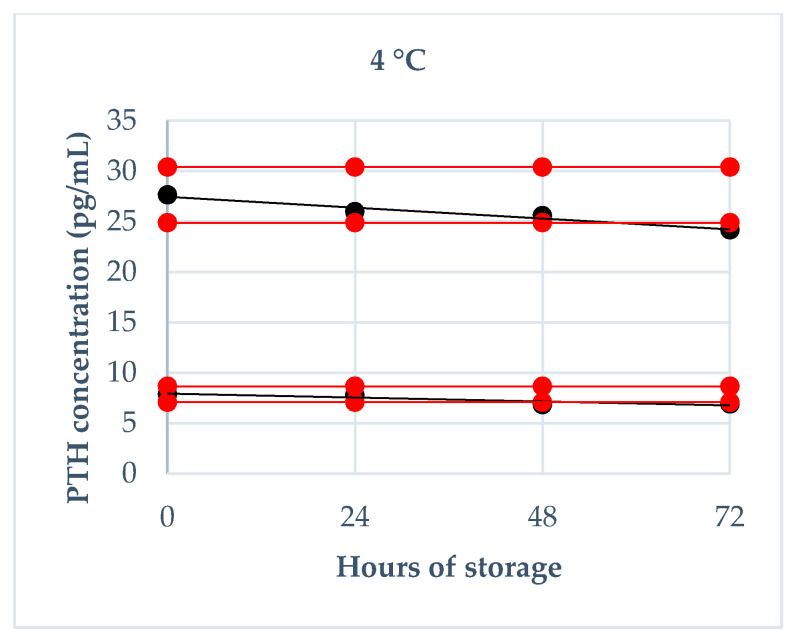
PTH stability at 4 °C during 72 h of storage (24 h, 48 h, 72 h): the black lines indicate the tendency of the obtained values; the red lines indicate ± 10% from the baseline value.

**Figure 4:**
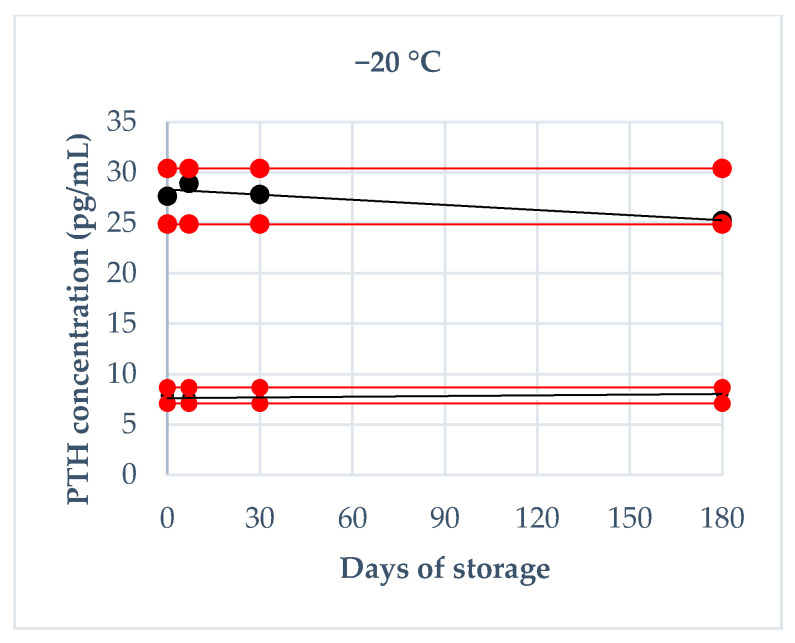
PTH stability at −20 °C during 180 d of storage (7 d, 30 d, 180 d): the black lines indicate the tendency of the obtained values; the red lines indicate ± 10% from the baseline value.

The results regarding the deviation from baseline values obtained on fresh serum at each storage time are reported in Figure 2, Figure 3 and Figure 4. Briefly, a deviation not exceeding 10% in both pools was obtained: at 20 °C within 4 h, at 4 °C within 24 h, at −20 °C after 6 months. Moreover, intra-assay CVs exceeded 7%: at room temperature after 24 h in both PTH pools and after 8 h only in low PTH pool; at refrigeration temperature after 72 h in both PTH pools and after 48 h only in low PTH pool; never at freezing temperature.

### 3.4. Clinical Validation

A total of 21 samples from 15 nephropatic dogs were obtained: sCr was available for all samples, while p was measured in 18 of 21. Median sCr was 2.96 mg/dl (0.9−18.06) and median *p* was 4.55 mg/dl (2.3–14.7).

**Figure 5:**
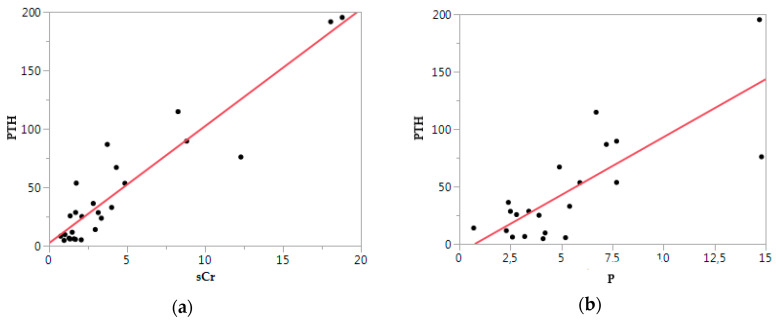
Parathyroid hormone (PTH) vs. biochemical parameters: (**a**) PTH vs. serum creatinine (sCr); (**b**) PTH vs. Phosphorus (P). Each point indicates a case. The red line is a tendency line.

PTH was significantly positively associated with sCr (*p* < 0.01, ρ = 0.93) and *p* (*p* < 0.01, ρ = 0.79), as reported in Figure 5. 

**Figure 6:**
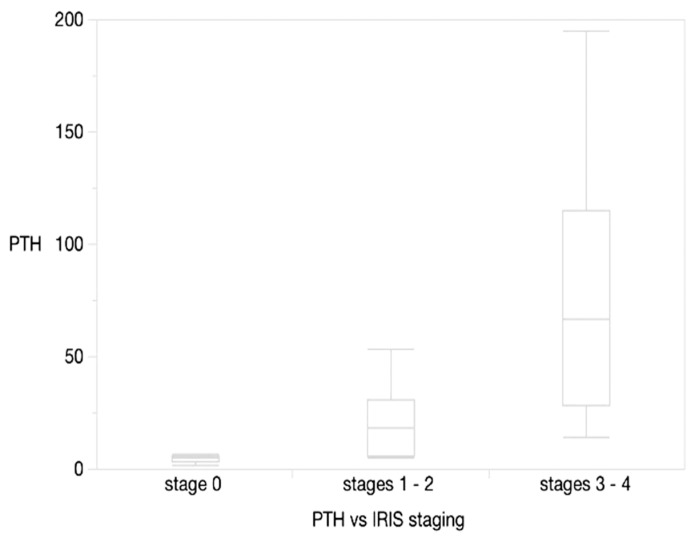
PTH vs. grouped IRIS stages. Horizontal lines represent median, boxes represent the interquartile range, whiskers extend to upper al lower limits.

PTH was also significantly different among IRIS stages, grouped as reported in Figure 6: 0 vs. 1–2 (*p* = 0.0168), 0 vs. 3–4 (*p* = 0.0022), 1–2 vs. 3–4 (*p* = 0.0067).

## 4. Discussion

PTH measurement is considered of clinical utility in dogs affected with any stage of CKD, in order to determine if hyperparathyroidism is present, since RHPT could occur at different stages of CKD, and also in the absence of hyperphosphatemia. Moreover, PTH determination is needed in the monitoring of the efficacy of the treatment for hyperphosphatemia or RHPT [2]. Several methods, some without sufficient validation with canine samples or not currently available, have been reported and used for the measurement of canine PTH and different ranges of values have been reported both in healthy and CKD dogs [3,4,7,8,12,13,14,15,17,18,23]. For these reasons, the validation in dogs of a new accessible method could be of clinical relevance.

In this study, a very satisfying precision and accuracy of the ST AIA-PACK^®^ Intact PTH assay in the detection of PTH in canine serum was demonstrated. Specifically, intra-assay CVs were always less than 7%, and inter-assay CVs were within 3.5%. When compared with chemiluminescent methods, very similar intra assay and lower inter-assay were obtained, suggesting a higher precision of the method investigated here; these values are slightly lower also if compared to whole and intact IRMA methods in dogs [16,19,24,25]. However, regardless of comparison with other methods, all CVs were significantly lower than 15%, which is considered acceptable for an immunoassay (Guideline on bioanalytical method validation, EMEA/CHMP/EWP/192217/2009 Rev. 1 Corr. 2, Committee for Medicinal Products for Human Use (CHMP, 2011, [26])), confirming optimal analytical performance. The accuracy estimated using SRT and LUD also showed satisfying results, with an optimal concordance between obtained and expected results. The limit of detection identified was about 1.3 pg/mL, which is higher than those obtained with IRMA (0.3 pg/mL) reported by Mooney et al. [16], but lower than those reported with some ELISA available for research purposes (Canine Intact PTH EIA MicroVue Bone (Quidel Corporation), Canine PTH ELISA kit (Elisagenie), Dog PTH ELISA kit competitive EIA (LifeSpan BioSciences)), which are near 3 pg/mL. However, although no Reference Intervals of the investigated method are available until now, the lower values obtained in healthy dogs reported in other studies, obtained with other methods, were always higher than 6 pg/mL. Moreover, as reported previously, in CKD dogs, PTH values increase according to the severity of the disease, reaching values above 100 pg/mL in some cases [3,4,7,13,17,23]. Consequently, the LLOD of our method was considered absolutely acceptable from a clinical point of view.

The evaluation of storage stability allowed us to evaluate the stability of the PTH at different temperatures. Firstly, it is interesting to underline that the immediate freezing maintains the concentration of the hormone for long times (until 6 months) without significant variation, irrespective of the concentration; this result could be useful from a research point of view. At room and refrigeration temperatures, the PTH determination should be done respectively within 4 h and 24 h in order to avoid misinterpretation; these times are compatible with the routine activity of a laboratory and allows the shipping to a reference laboratory without relevant hormone concentration decrease. These results are quite similar to those reported in human studies (stability of 2 h at room temperature and 24 h at 4 °C), even if data are often contradictory and vary between studies and methods [27].

Currently, a gold standard method for the measurement of PTH in dogs is not easy to identify because of the reported limitations in all the methods employed. Although IRMA are often considered a reference technique for hormones determination, some methods are currently difficult to access [16,28]. Moreover, the best method for the measurement of PTH in CKD patients still remains a challenging issue also in human medicine, even if the Kidney Disease: Improving Global Outcomes guidelines (2017) suggest the use of a second-generation assay, as the investigated method [6,29]. For these reasons, a clinical validation using was planned in order to demonstrate the usefulness of the PTH measurement in CKD dogs through ST-AIA Pack^®^ Intact PTH. Although we consider that this part of the study is preliminary and limited, the obtained associations with sCr, P and the significant differences detected in the grouped IRIS stages are in concordance with reported data and ensure the legitimacy of our validation [3,4,13,17]. Phosphorus retention and consequently hyperphosphatemia are the main promoters in the pathophysiological cascade that lead to the increase of PTH through the interaction between iCa, P, PTH, FGF-23 and vitamin D metabolites and the association between PTH and phosphatemia agrees with the mechanism of the RHPT [2]. RHPT is frequent in CKD dogs and associated with the severity of the disease and is often present before the presence of hyperphosphatemia [3]. In fact, if it is not unexpected the significant difference between healthy and advanced stages of CKD, it is more interesting the detection of higher values in early stages (IRIS stages 1 and 2) compared to stage 0. These data need further investigations because the early identification of RHPT in CKD could have a significant impact on the therapeutic approach [5]. 

## 5. Conclusions

This study demonstrates that ST-AIA PACK^®^ Intact PTH, even if based on human antibodies, is a precise and accurate immunoenzymometric assay in the detection of PTH in canine serum, and could be available in reference laboratories for clinical purposes. The lower limit of detection of the method, equal to 1.3 pg/mL, is considered satisfying from a clinical point of view. 

The storage stability allows to reliable to be obtained results without significant variations within 4 h at room temperature and within 24 h at refrigeration temperature. Moreover, storage at freezing temperature preserves the concentration of PTH until 6 months. 

In absence of comparison with a reference method, the clinical validation obtained measuring PTH in healthy dogs and dogs affected with different degrees of CKD showed results consistent with the clinical status. PTH was significantly associated with sCr and phosphorus, and increased according to IRIS stage, suggesting the possible occurrence of renal hyperparathyroidism also in early stages of CKD in dogs.

Further studies are needed for the establishment of the reference intervals of the method in dogs and for better characterize the complex interactions between PTH and renal disease in a large population of spontaneously affected dogs.

## Figures and Tables

**Table 1:** Intra- and inter-assay imprecision calculated using pooled sera with low, medium and high Parathyroid Hormone concentration (pg/mL). SD: Standard Deviation; CV: Coefficient of Variation.

Parameter	Low	Medium	High
INTRA-ASSAY			
Mean	3.52	29.13	95.15
SD	0.23	0.77	5.68
CV	6.59	2.66	5.97
INTER-ASSAY			
Mean	3.67	28.41	96.5
SD	0.13	0.71	1.76
CV	3.42	2.51	1.83

**Table 2:** Spike Recovery Test: comparison between expected and obtained results (pg/mL).

Recovery Percentage	Expected	Obtained	BIAS	%
100%	3.5	3.5	0	0
90%	10.54	10.8	0.26	2.5
80%	17.58	18.3	0.72	4.1
70%	24.62	26	1.38	5.6
60%	31.66	31.8	0.14	0.4
50%	38.7	40.4	1.7	4.4
40%	45.74	45	−0.74	−1.6
30%	52.78	52.6	−0.18	−0.3
20%	59.82	61.4	1.58	2.6
10%	66.86	67.9	1.04	1.6
0%	73.9	73.9	0	0

## Supplementary Materials

The following are available online at https://www.mdpi.com/2076-2615/10/12/2411/s1, Table S1. Manuscript Analytical and clinical validation of a new immunoenzymatic method for the measurement of canine parathyroid hormone Supplementary.
