# Supplementary material for: Analytical and Clinical Validation of a New Immunoenzymatic Method for the Measurement of Canine Parathyroid Hormone
Source: Animals (Basel). 2020 Dec 17;10(12):2411. doi: 10.3390/ani10122411 (PMC7766972; doi:10.3390/ani10122411)
Supplement: Supplementary file 1 [file animals-10-02411-s001.pdf]

animals

MDPI

Article

Analytical and Clinical Validation of a New Immunoenzymatic Method for the Measurement of Canine Parathyroid Hormone

Jari Zambarbieri, Filippo Tagliasacchi, Pierangelo Moretti, Alessia Giordano [1] and Paola Scarpa

Table S1. Manuscript Analytical and clinical validation of a new immunoenzymatic method for the measurement of canine parathyroid hormone Supplementary Materials.

ID	BREED	SEX	AGE	GROUP	IRIS STAGE	PTH (pg/ml)	sCr (mg/dl)	TOTAL CALCIUM (mg/dl)	INORGANIC PHOSPHORUS (mg/dl)
1	GOLDEN RETRIEVER	NF	7	CKD	2	6	1.6	10.5	2.6
2	MONGREL	NF	16	CKD	2	25	2.1	11.1	3.9
3	GERMAN SHEPHERD	F	2	CKD	4	114.7	8.27	9	6.7
4	GERMAN SHEPHERD	F	2	CKD	4	89.5	8.82	11.7	7.7
5	GERMAN SHEPHERD	F	2	CKD	4	195.3	18.8	10.2	14.7
6	GERMAN SHEPHERD	F	2	CKD	4	191.6	18.06		
7	WELSH CORGI PEMBROKE	M	1	CKD	2	53.6	1.74	11.7	7.7
8	MONGREL	NF	15	CKD	2	28.5	1.7	11.3	3.4
9	FOX TERRIER	NF	9	CKD	1	9.6	1.01	10.6	4.2
10	GOLDEN RETRIEVER	F	1	CKD	2	5.5	1.69	11.3	5.2
11	MONGREL	F	11	CKD	3	32.8	4.01	11.4	5.4
12	BOXER	NF	9	CKD	1	25.6	1.35	10.5	2.8
13	LABRADOR RETRIEVER	F	7	CKD	3	67	4.31	10.1	4.9
14	LABRADOR RETRIEVER	F	7	CKD	3	53.4	4.86	11.3	5.9
15	AMERICAN AKITA	F	1	CKD	2	5.1	2.06		
16	GOLDEN RETRIEVER	F	5	CKD	3	13.9	2.96	11.3	2.7

17	GOLDEN RETRIEVER	F	5	CKD	3	28.4	3.15	11.1	2.5
18	GOLDEN RETRIEVER	F	5	CKD	3	23.6	3.35		
19	LABRADOR RETRIEVER	NF	11	CKD	2	11.6	1.48	10.6	2.3
20	MONGREL	NF	10	CKD	2	36.2	2.83	10.5	2.4
21	MALTESE	NM	11	CKD	3	86.7	3.73	9.8	7.2
22	ENGLISH COCKER SPANIEL	NM	3	HEALTHY	0	6.5	0.74		
23	SPRINGER SPANIEL	NF	14	HEALTHY	0	4.6	1.28	10	3.2
24	GREAT DANE	M	5	HEALTHY	0	5.8	1.34		
25	MONGREL	NF	11	HEALTHY	0	1.8	0.9	10.9	2.6
26	MONGREL	NM	1	HEALTHY	0	4.6	0.96	9.8	4.1
